# Diabetes is associated with increased and persistent myocardial edema in infarct segment post acute myocardial infarction

**DOI:** 10.1186/1532-429X-14-S1-P29

**Published:** 2012-02-01

**Authors:** Mohammad I Zia, Nilesh R Ghugre, Kim A Connelly, Bradley H Strauss, Alexander J Dick, Graham Wright

**Affiliations:** 1 Sunnybrook Health Sciences Centre, Toronto, ON, Canada

## Summary

Our study demonstrates that diabetic patients have increased myocardial edema in the infarct segment at an early time point post acute myocardial infarction which persists till the subacute phase. This may lead to deleterious left ventricular remodeling and worse outcome in these patients.

## Background

Diabetes is associated with worse left ventricular remodeling and poor prognosis in patients post acute myocardial infarction (AMI). The impact of diabetes on microvascular injury parameters including myocardial edema and hemorrhage post AMI is unknown. Our objective was to characterize the evolution of myocardial edema and hemorrhage post AMI in patients with and without diabetes.

## Methods

Sixty patients were enrolled post AMI and underwent cardiac magnetic resonance on a GE Signa Excite, 1.5T scanner with a 8-channel receive coil at 48 hours and 3 weeks. T2 maps were computed from a previously validated cardiac-gated spiral imaging sequence with T2 preparations yielding TEs=2.9,24.3,88.2,184.2ms to assess myocardial edema. The T2* sequence was a multiecho acquisition with 8 echoes (between 1.4 and 12.7ms) acquired at TR=14.6ms. Delayed hyperenhancement was also performed. We retrospectively reviewed and stratified patients into those that did and did not have diabetes (type 1 or type 2).

## Results

We compared 15 diabetics versus 45 non-diabetics (Table 1). Baseline characteristics including age, gender, symptom to balloon time, door to balloon time, glycoprotein IIb/IIIa inhibitor use, and thrombus aspiration use were similar in both groups. The mean T2 was higher in the infarct segment (IS) compared to remote segment (RS) in both patient groups (diabetics: 59.3ms vs 41.3ms, p<0.0001 vs non-diabetics: 52.9ms vs 40.1ms, p<0.0001). The mean T2* was similar in the IS compared to RS in both diabetics (32.1ms vs 38.0ms; p=0.11) and non-diabetics (33.9ms vs 36.7ms; p=0.09). The mean T2 was significantly higher in the IS of diabetic patients versus non-diabetics (59.3ms vs 52.9ms; p=0.03). The mean T2* was equivalent in the IS of both patient groups (32.1ms vs 33.9ms; p=0.59).

**Table 1:** 

Baseline characteristic	Diabetic (n=15)	Non-diabetic (n=45)
Mean age, years	59.5	59.9
Male, %	2 (13)	5 (11)
Anterior MI, %	7 (47)	19 (42)
Mean peak CK, IU/L	2521	2134
Symptom to balloon time, minutes	452	373
Door to balloon time, minutes	93.1	79.8
LVEF, %	45.3	44.9
Infarct size, %	23.2	22.5

At 3 weeks, the mean T2 remained significantly higher in the IS of diabetic patients versus non-diabetics (56.6ms vs 51.2ms; p=0.04). At this time interval, the mean T2* was equivalent in the IS of both patient groups (37.1ms vs 37.0ms; p=0.96).

## Conclusions

Our study demonstrates that diabetic patients have increased myocardial edema in the IS at an early time point post AMI which persists till the subacute phase (3 weeks). The degree of myocardial hemorrhage does not appear to be influenced by diabetes status. Diabetes increases the area at risk during AMI which may lead to deleterious left ventricular remodeling and worse outcome in these patients.

## Funding

Dr. Connelly is supported by a Heart and Stroke Foundation of Canada Phase 1 Clinician Scientist Award. Dr. Wright receives research funding from GE Healthcare. Dr. Dick is supported by the Heart and Stroke Foundation of Canada. This work was supported in part by a Canadian Institutes of Health Research (CIHR) operating grant and the Ontario Research Fund.

